# Does the length of uniportal video‐assisted thoracoscopic lobectomy affect postoperative pain? Results of a randomized controlled trial

**DOI:** 10.1111/1759-7714.13291

**Published:** 2020-05-07

**Authors:** Cecilia Menna, Camilla Poggi, Claudio Andreetti, Giulio Maurizi, Anna Maria Ciccone, Antonio D'Andrilli, Camilla Vanni, Anna Rita Vestri, Alfonso Fiorelli, Mario Santini, Federico Venuta, Erino Angelo Rendina, Mohsen Ibrahim

**Affiliations:** ^1^ Division of Thoracic Surgery, Sant'Andrea Hospital University of Rome “Sapienza” Rome Italy; ^2^ Division of Thoracic Surgery, Policlinico Umberto I University of Rome “Sapienza” Rome Italy; ^3^ Department of Public Health and Infectious Disease University of Rome “Sapienza” Rome Italy; ^4^ Thoracic surgery Unit University of Campania Luigi Vanvitelli Naples Italy; ^5^ Fondazione Eleonora Lorillard Spencer Cenci Rome Italy

**Keywords:** Lobectomy, post‐operative pain, uniportal video‐assisted thoracoscopic surgery

## Abstract

**Background:**

Uniportal video‐assisted thoracoscopic surgery (VATS) lobectomy has become a common approach for the treatment of early stage lung cancer. Here, we aimed to establish whether the length of uniportal incision could affect postoperative pain and surgical outcomes in consecutive patients undergoing uniportal VATS lobectomy for early stage lung cancer.

**Methods:**

This was a unicenter Randomized Control Trial (NCT 03218098). Consecutive patients undergoing uniportal VATS lobectomy for Stage I lung cancer were randomly assigned to a Small Incision group or Long Incision group in 1:1 ratio based on whether patients received a 4 cm or 8 cm incision. The endpoints were to compare the intergroup difference regarding (i) postoperative pain measured by brief pain inventory (BPI) questionnaire (first endpoint); (ii) operative time; (iii) length of chest drainage; (iv) length of hospital stay; (v) postoperative complications; and (vi) pulmonary functional status (secondary endpoints).

**Results:**

A total of 48 patients were eligible for the study. Four patients were excluded; the study population included 44 patients: 23 within the Small Incision group, and 21 within the Long Incision group. The 11 BPI scores between the two groups showed no significant difference. Small Incision group presented higher operative time than Long Incision group (138.69 vs. 112.14 minutes; *P* = 0.0001) while no significant differences were found regarding length of hospital stay (*P* = 0.95); respiratory complications (*P* = 0.92); FEV1% (*P* = 0.63), and 6‐Minute Walking Test (*P* = 0.77).

**Conclusions:**

A larger incision for uniportal VATS lobectomy significantly reduced the operative time due to better exposure of the anatomical structures without increasing postoperative pain or affecting the surgical outcome.

**Key points:**

- A larger incision for uniportal VATS lobectomy significantly reduced the operative time due to better exposure of the anatomical structures without increasing postoperative pain or affecting the surgical outcome.
- To perform a larger incision could be a valuable strategy, particularly in nonexpert hands or when the patient's anatomy or tumor size make exposure of anatomic structures through smaller incisions difficult.

## Introduction

In the last decade, video‐assisted thoracoscopic surgery (VATS) lung resection has become the preferred approach among most thoracic surgeons for the management of early stage lung cancer. The reduction of postoperative pain and better surgical outcomes are the main advantages of VATS compared to standard thoracotomy.^1^, ^2^, ^3^, ^4^ Uniportal VATS (UVATS) represents a further evolution of standard multiports VATS.^5^ During UVATS, surgical instruments are introduced parallel to video‐thoracoscopy through a single incision, without rib spreading. The operative view is similar to standard thoracotomy, whilst preserving the advantages of minimally invasive surgery. However, there is no standardization on the length of the UVATS incision in the literature as it depends on several variables, such as the type of resection, patient anatomy and capability of the surgeon. A larger UVATS incision facilitates surgical maneuverability, especially in nonexpert hands, but can also increase postoperative pain and surgical outcome in comparison to a smaller incision.

Thus, the aim of this paper was to establish whether the length of a uniportal incision could affect postoperative pain and surgical outcomes in consecutive patients undergoing UVATS lobectomy for early stage lung cancer.

## Methods

### Study design

This was a Randomized Control Trial (RCT) conducted at the Thoracic Surgery Unit of Sapienza University, Rome, Italy, from January 2014 to January 2016. Consecutive patients undergoing UVATS lobectomy for stage I lung cancer were randomly assigned to a Small Incision (SI) group or Long Incision (LI) group in 1:1 ratio based on whether patients received a 4 cm or a 8 cm incision. There were no significant changes to the trial methodology after trial commencement with regard to the type of randomization or eligibility criteria.

The study design, planned according to the CONSORT‐SPIRIT guideline,^6^ was approved by the Local Ethical Committee of Sapienza University (approval code number: 1037/2012), and registered at ClinicalTrial.gov (NCT 03218098). Participants were informed that their participation was voluntary and that they could withdraw consent to participate at any time during the study without any consequences for their care. All patients gave a written informed consent before entering into the study.

### Participants in the study

Inclusion criteria were: (i) patients were age ≥ 18 years (of either gender); (ii) lobectomy had been performed using UVATS; (iii) written informed consent had been received prior to participation in the study. Exclusion criteria were: (i) a history of previous thoracotomy; (ii) chronic pain syndrome (any type of pain), opioid/steroid use six months before surgery, or chest trauma with rib fractures; (iii) radiologic or surgical evidence of pleural adhesions; (iv) participation in another interventional clinical trial, or undergoing a surgical procedure within the last 30 days; and (v) conversion to thoracotomy.

### Surgical procedure

**Figure 1:**
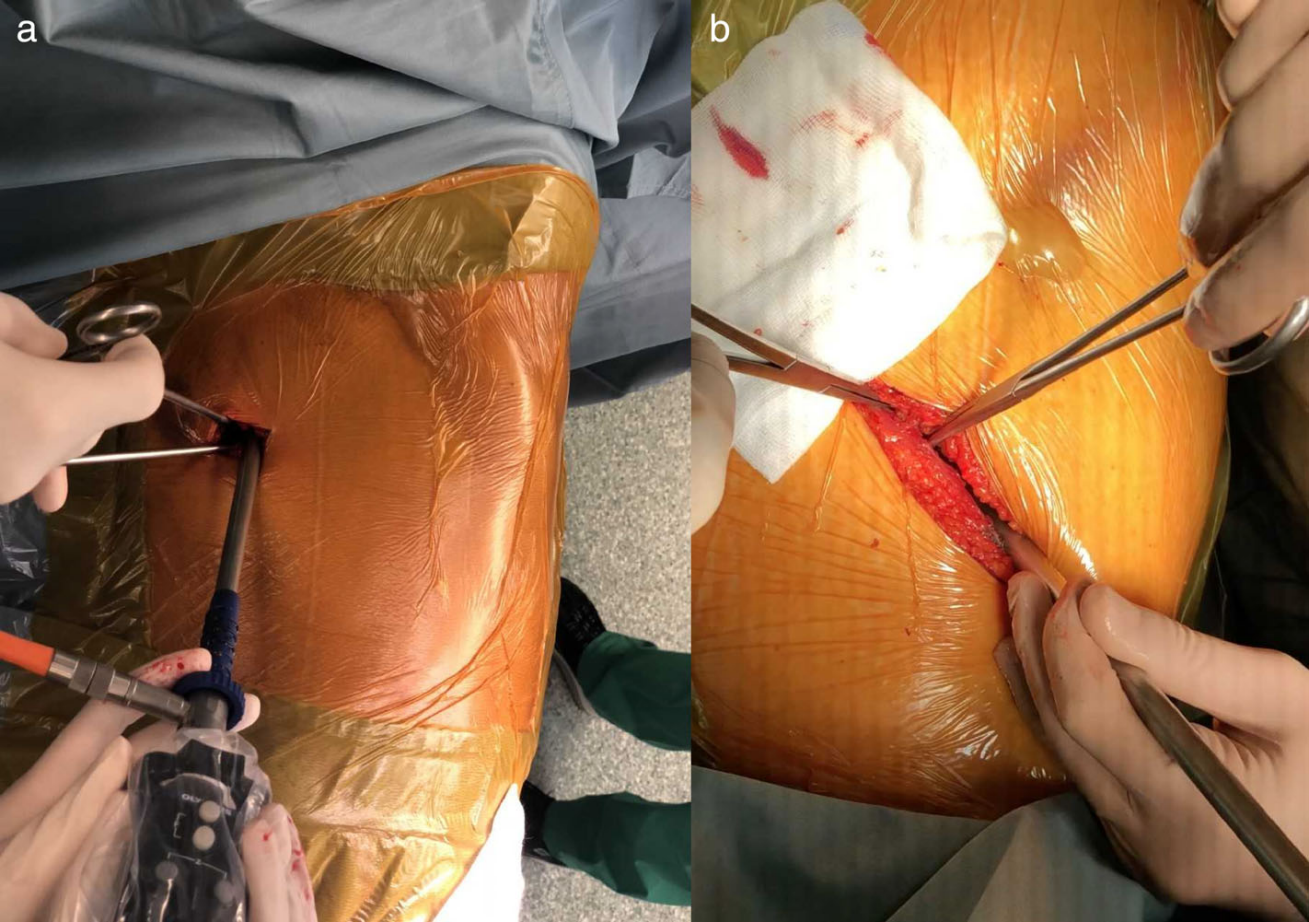
Different lengths of uniportal incision: 4 cm (Small Incision group, Part a) or 8 cm (Long Incision group, Part b).

All procedures were performed under general anesthesia with single lung ventilation. Both surgeon and assistant stood anteriorly facing the patient and a second assistant stood posteriorly. A single incision of approximately 4 cm (SI group, Fig. 1a) or 8 cm (LI group, Fig. 1b) long depending on the allocated group was performed at the fifth intercostal space along the anterior axillary line, anteriorly to the latissimus dorsi muscle (muscle‐sparing technique). Trocars, rib retractors, and soft tissue separators were not used. The video‐thoracoscope (30° 5 mm) and multiple VATS instruments were simultaneously inserted into the uniport. In order to avoid the overlap of multiple instruments (that may strike the thoracoscopy and result in shaky movements on the video monitor), the scope was placed at the posterior angle of thoracotomy. Long and curved instruments were used to allow easy simultaneous insertion of two or three devices. No additional skin incisions were made. When the pulmonary artery was not visible in the fissure, the procedure was performed from bottom to top, with fissure stapling as the final step. Hilar structures were approached anteriorly in the same sequence as traditional VATS lobectomy (pulmonary artery, pulmonary vein, and bronchus). Vessels were individually mechanically stapled, as well as the lobar bronchus (ECHELON ENDOPATH, Ethicon Endo‐Surgery, Norderstedt, Germany). The resected lobe was retrieved using a specimen bag (ENDOCATCH, Covidien, La Ciotat, France) through the same incision. Systematic mediastinal lymph node dissection was routinely performed. For paratracheal and subcarinal dissection it was very helpful to have the patient in the reverse‐Trendelenburg position. Placing the camera in the upper part of the incision and inserting three or four instruments below the camera to complete the systematic dissection of the subcarinal space and paratracheal on the right side and subcarinal and aorto‐pulmonary window on the left side was necessary. For right subcarinal lymph node dissection, the esophagus and intermediate bronchus were separated to facilitate the procedure. For paratracheal lymph node dissection, the procedure was carried out by opening the pleura inferiorly to the azygos vein, lifting the azygos vein and retracting the superior vena cava to the right side. For hilar and N1 station lymphadenectomy, the operating table was moved and rotated posteriorly in order to place the lung in the back position. The intercostal muscle was approximated by fixing it to the periosteal tissue. At the end of the operation, a 20 Ch chest tube was inserted at the posterior extremity of the thoracotomy.

Pain management was ensured with a multilevel intercostal nerve block using ropivacaine (7.5 mg diluted in 20 mL of saline), 4 mL for each intercostal space, including the intercostal level of the incision and one level above and below. Baseline analgesia for all patients consisted of continuous intravenous infusion of Tramadol (10 mg/hour) and ketorolac tromethamine (3 mg/hour) beginning at UVATS and continuing until 48 hours after surgery. Subsequent analgesia was administered according to patient request.

### Primary endpoint

In order to evaluate and compare postoperative pain among the two study groups, the pain level was evaluated at fixed times: one hour, 24 hours, 48 hours, and one month postoperatively. Patients subjectively evaluated their pain using the Brief Pain Inventory (BPI). The BPI is a questionnaire that rapidly assesses the severity of pain and its impact on daily functions. Patients separately rated, using the same type of scale, the four pain severity items and seven pain interfering items, including enjoyment of life, general activity, walking, mood, sleep, work, and relationship with others. These items were 0 = does not interfere and 10 = interferes completely. The arithmetic mean of the scores was used as a pain interference score.

### Secondary endpoint

The secondary endpoints were to evaluate and compare the following variables in both study groups: (i) operative time was defined as the time taken from skin incision to completion of skin closure; (ii) length of chest drainage was calculated from the day of tube insertion until its removal (days) (iii) length of hospital stay was calculated from the day of the operation until discharge (days) (iv) postoperative complications. Operative mortality was defined as any death within 30 days of operation or prior to dismissal. Complications were classified as major (potentially life‐threatening) including pneumonia, myocardial infarction, stroke, bleeding, adult respiratory failure, and need for reintubation occurring within 30 days of surgery, or within three months in cases of empyema or bronchopleural fistula; and as minor (nonlife‐threatening, requiring medical therapy and/or prolonged hospital stay and (v) pulmonary functional status. Flow expiratory volume in one second (FEV1%) expressed as a percentage of predicted value and six‐minute walking test (6‐MWT) were measured before the operation and on discharge.

### Sample size

Group sample sizes of 20 and 20 achieved 90% power to detect a difference of 28.0 between the null hypothesis that both group means were 140.0 and the alternative hypothesis that the mean of group 2 was 112.0 with known group standard deviations of 33.0 and 20.0 and with a significance level (alpha) of 0.05 using a two‐sided two‐sample *t*‐test. A drop‐out rate of approximately 20% was considered, and the established total sample size was 48 patients.

### Randomization

Participants were randomly assigned with a 1:1 allocation to SI group or LI group based on computer‐generated codes. Each patient had an equal probability of being assigned to either the SI group or LI group. The assignments were kept in sealed envelopes and the operating surgeon ascertained the treatment allocation for each eligible patient by opening the sealed envelopes one hour before the operation. All investigators except for the operating surgeon were blind to the patient's assigned group.

### Statistical analysis

Data were summarized as mean and standard deviation (SD) for normally distributed continuous variables; and absolute number and percentage for categorical variables, as appropriate. For the analysis of quantitative variables (normality of distribution verified by the Kolmogorov‐Smirnov test), *t*‐test was used; Fisher's test was used to compare qualitative variables. Repeated measures analysis of variance (ANOVA) was performed to assess differences in pain intensity scores and in pain interference scores across time and between treatment groups, and for the interaction between time and treatment group. The difference was considered significant for *P*‐values ≤0.05. All analysis was performed with SPSS software (version 13.0, SPSS, Chicago, IL, USA).

## Results

**Figure 2:**
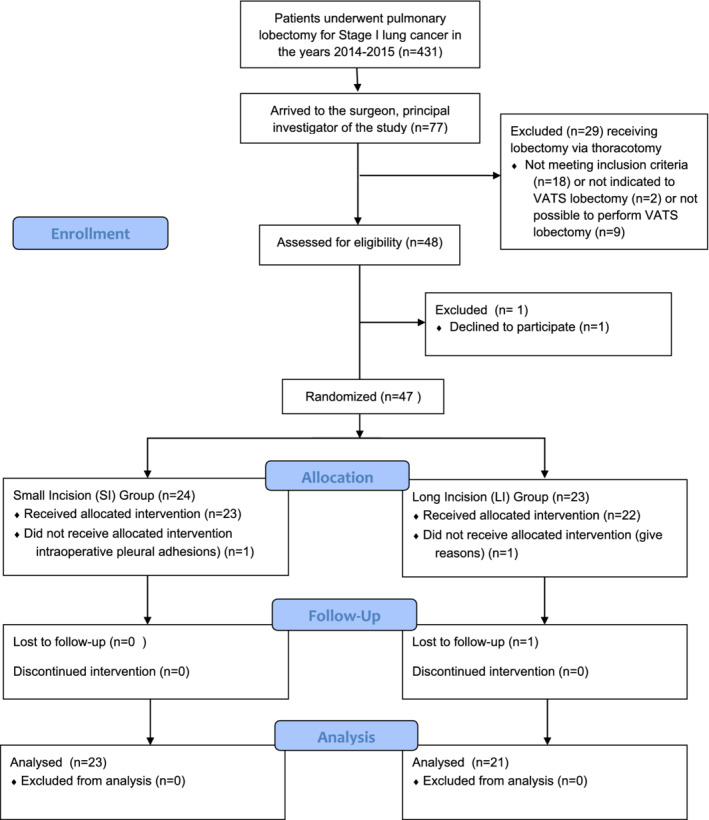
Flow chart of study according to CONSORT‐SPIRIT guideline.

A total of 48 patients were eligible inclusion in the study. One patient declined to participate, thus 47 patients were randomized to the SI group (*n* = 23) and to the LI group (*n* = 24). Among these, a patient of SI group and a patient of LI group were excluded due to conversion to thoracotomy as a result of the presence of pleural adhesions. Also, a patient in the LI group did not complete follow‐up. Thus, our study population consisted of 44 patients (23 patients in the SI group, and 21 patients in the LI group). The flow diagram of study population, planned according to the CONSORT‐SPIRIT guideline, is summarized in Fig. 2. The comparison of both study groups showed no significant difference in terms of demographic variables, preoperative comorbidity, type of surgical procedure and histology (Table 1).

**Table 1:** Clinical characteristics of patients

Variable	SI group (*n* = 23)	LI group (*n* = 21)	*P*‐value
Sex, n (%)			
Male	14 (60.9)	15 (71.4)	0.46
Female	9 (39.1)	6 (28.6)	0.095
Age (mean/range/SD, years)	65.2/56–83/8.6	67.2/46–86/14.3	0.54
COPD, n (%)	3 (13.0)	1 (4.8)	0.34
Preoperative FEV1%	88.1 ± 14.0	83.2 ± 9.1	0.93
Preoperative 6‐MWT (metres)	207.1 ± 75.9	214.2 ± 69.5	0.22
Surgery, n (%)			
‐Right upper lobectomy	6 (26.1)	5 (23.8)	0.86
‐Middle lobe lobectomy	1 (4.3)	2 (9.5)	0.49
‐Right lower lobectomy	3 (13.0)	4 (19.0)	0.58
‐Left upper lobectomy	7 (30.4)	6 (28.6)	0.89
‐Left lower lobectomy	6 (17.4)	4 (19.0)	0.57
Histology, n (%)			
Adenocarcinoma	16 (69.6)	13 (61.9)	0.44
Squamous cellcarcinoma	4 (17.4)	6 (28.6)	0.37
Other	3 (13.0)	2 (9.5)	0.71

COPD, chronic obstructive pulmonary disease; LI, Long Incision; SD, standard deviation; SI, Small Incision.

### Primary endpoint

The results are summarized in Table 2. The 11 BPI items and differences of mean postoperative pain scores and pain interference scores between the two groups showed no significant difference. Doses and frequency of additional analgesia administration were recorded and compared between the two groups with no differences in analgesia intake. After chest tube removal and hospital discharge, no patient required more than paracetamol (maximum 3 g per day) to manage their postoperative pain.

**Table 2:** Pain results

	24 hours			48 hours			1One month		
Variable	SI (*n* = 23)	LI (*n* = 21)	*P*‐value	SI (*n* = 23)	LI (*n* = 21)	*P*‐value	SI (*n* = 23)	LI (*n* = 21)	*P*‐value
Pain on average	4.1 ± 0.8	4.2 ± 0.7	0.32	3.1 ± 0.6	3.2 ± 0.4	0.42	0.3 ± 0.7	0.4 ± 0.4	0.51
Maximum pain	6.5 ± 1.2	6.0 ± 1.1	0.48	4.0 ± 0.3	4.4 ± 0.4	0.37	0.9 ± 0.6	0.3 ± 0.6	0.36
Minimum pain	3.4 ± 0.8	3.2 ± 0.7	0.62	0.6 ± 0.9	0.7 ± 0.6	0.29	0.1 ± 0.1	0.2 ± 0.2	0.24
Actual pain	4.7 ± 0.9	4.3 ± 0.8	0.49	1.6 ± 0.7	1.8 ± 0.8	0.43	0.2 ± 0.5	0.2 ± 0.4	0.29
Interference general activity	5.0 ± 1.3	5.3 ± 0.8	0.34	4.1 ± 0.7	3.9 ± 0.9	0.42	0.2 ± 0.3	0.2 ± 0.5	0.42
Interference mood	5.6 ± 0.7	5.7 ± 0.6	0.24	1.3 ± 0.7	1.4 ± 0.5	0.62	0.3 ± 0.8	0.2 ± 0.6	0.41
Interference walking ability	4.1 ± 0.8	4.0 ± 0.5	0.43	2.1 ± 0.9	1.9 ± 1.6	0.47	0.1 ± 0.5	0.1 ± 0.3	0.39
Interference relationships	2.8 ± 1.1	2.6 ± 1.4	0.32	1.6 ± 1.0	1.9 ± 0.2	0.51	0.1 ± 0.4	0.2 ± 0.3	0.29
Interference enjoyment of life	5.7 ± 0.9	5.1 ± 0.3	0.45	2.4 ± 0.8	2.3 ± 0.5	0.37	0.3 ± 0.2	0.2 ± 0.8	0.47
Interference sleep	6.4 ± 1.6	6.9 ± 0.5	0.37	3.6 ± 1.9	3.3 ± 1.6	0.43	0.2 ± 0.3	0.1 ± 0.6	0.49
Interference working	6.6 ± 1.4	6.4 ± 0.9	0.24	4.7 ± 1.3	3.8 ± 0.9	0.36	0.7 ± 0.8	0.6 ± 0.4	0.53

All values are expressed in mean ± SD. SD, standard deviation.

### Secondary endpoint

Postoperative outcomes are summarized in Table 3. Mean operative time in the SI group was 138.7 ± 23.1 min and 112.1 ± 12.3 min in the LI group (*P* = 0.0001). Median duration of chest tube permanence was three days in the SI group and three days in the LI group (*P* = 0.81). Respiratory complications (pulmonary atelectasis and slow pulmonary re‐expansion) occurred in two patients (8.69%) in the SI group and two patients (9.5%) in the LI group (*P* = 0.924). No deaths occurred. Median length of hospital stay was three days for the SI group (range 2–5) and three days for the LI group (range 2–4, *P* = 0.924).

**Table 3:** Postoperative outcomes

Variable	SI group (*n* = 23)	LI group (*n* = 21)	*P*‐value
Operative time (mean ± SD, minutes)	138.7 ± 23.1	112.1 ± 12.3	0.0001
Length of hospital stay (median [range], days)	3 [2–5]	3 [2–4]	0.95
Respiratory complications, *n* (%)	2 (8.7%)	2 (9.5%)	0.92

SD, standard deviation.

**Figure 3:**
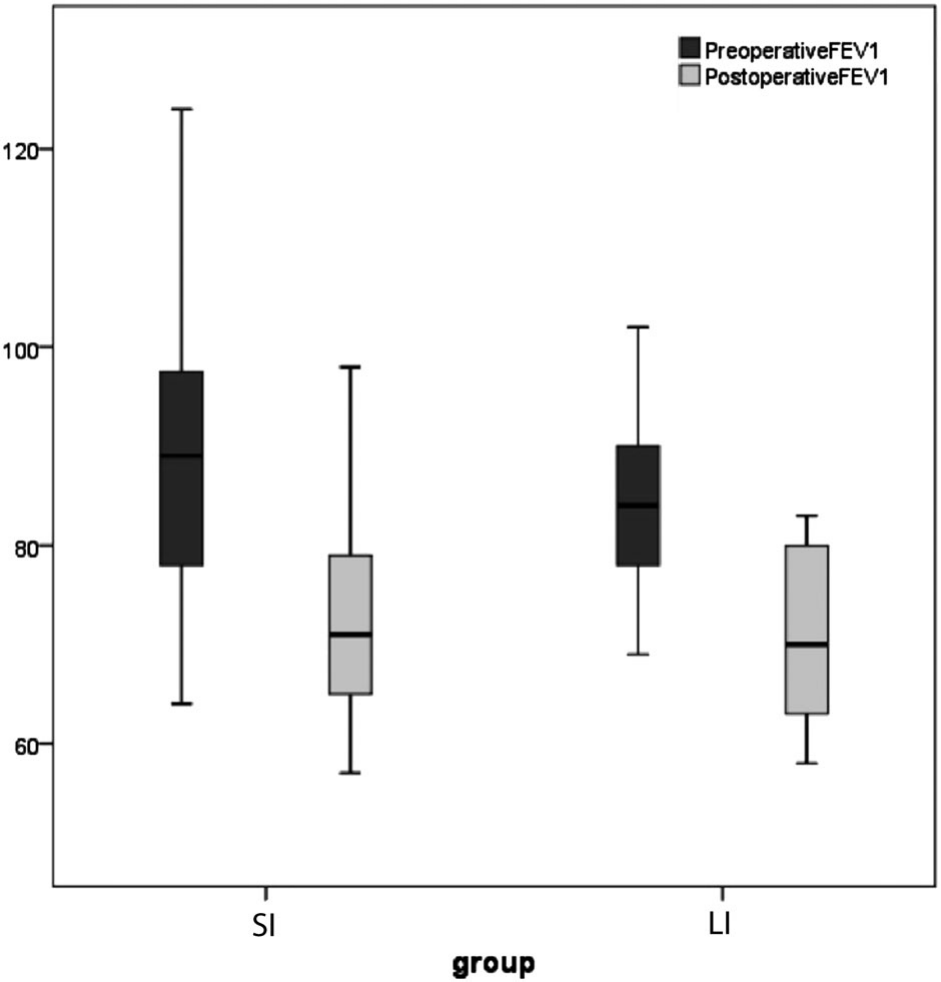
Comparison of pre and postoperative FEV1% in Small Incision (SI) and Long Incision group (LI).

**Figure 4:**
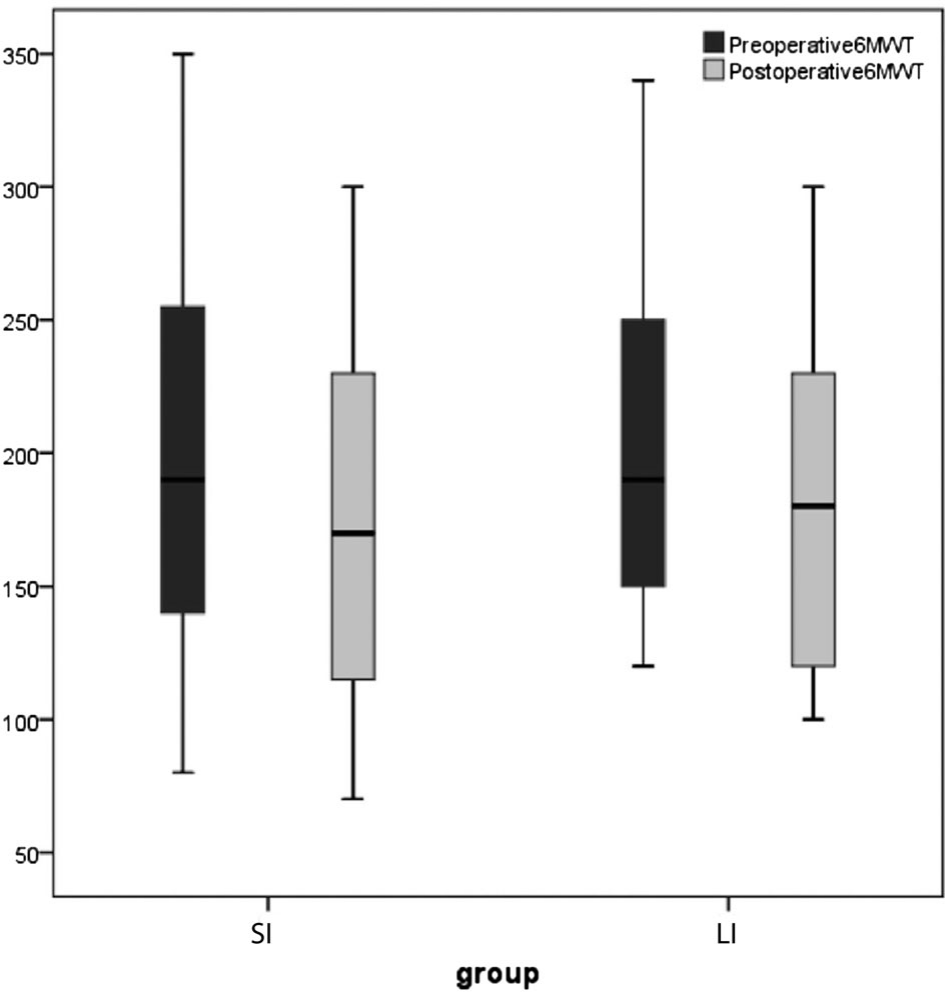
Comparison of preoperative and postoperative 6 MWT in Small Incision (SI) and Long Incision (LI) group.

Pulmonary and functional tests are summarized in Table 4. There were no significant differences among the two study groups before and after surgery. Preoperative FEV1% (Fig. 3) was 87.6 ± 14.1 L in the SI group and 83.7 ± 9.2 in the LI group (*P* = 0.45); postoperative FEV1% was 72.0 ± 10.2 in the SI group, and 70.8 ± 8.7 in the LI group (*P* = 0.36); 6‐MWT (Fig. 4) was 202.9 ± 74.9 in the SI group, and 209.0 ± 69.4 in the LI group (*P* = 0.68); postoperative 6‐MWT value was 172.4 ± 66.8 in the SI group, and 176.2 ± 61.9 M in the LI group (*P* = 0.77).

**Table 4:** Functional results

Variable	SI group (*n* = 23)	LI group (*n* = 21)	*P*‐value
Preoperative FEV1% (mean ± SD)	87.6 ± 14.1	83.7 ± 9.2	0.45
Postoperative FEV1% (mean ± SD)	72.0 ± 10.2	70.8 ± 8.7	0.36
Preoperative 6‐MWT (mean ± SD, m)	202.9 ± 74.9	209.0 ± 69.4	0.68
Postoperative 6‐MWT (mean ± SD, m)	172.4 ± 66.8	176.2 ± 62.0	0.77

FEV1%, forced expiratory volume in one second; 6‐MWT,sixminute walking test; SD, standard deviation.

## Discussion

Postoperative pain control remains a crucial problem in patients followinglung resection.^7^, ^8^ Pain impairs coughing and secretion clearing, resulting in bronchial obstruction and possible pulmonary infections. Over the years, minimally invasive procedures such as multiport VATS or UVATS have been preferred to thoracotomy for performing lung resection by most thoracic surgeons. These strategies are associated with a significant reduction in postoperative pain, morbidity and mortality compared to thoracotomy whilst still achieving the same oncological results.^1^, ^2^, ^3^, ^4^ During UVATS, the camera and surgical instruments are inserted through the same incision, allowing a direct view of lung lesions, without a horizontal plane or triangulation as in conventional VATS. The approach of the target lesion is similar to that in open surgery, with the operative fulcrum inside and not outside the chest.^9^, ^10^ There is currently no standardization of UVATS incision length reported in the literature. In theory, a larger UVATS incision facilitates surgical resection in comparison to a smaller incision, but it may be also associated with an increase in postoperative pain and a worse surgical outcome. To evaluate this issue for the first time we prospectively compared post‐operative pain and surgical outcomes in relation to the length of a UVATS incision.

First, our study showed that a larger UVATS incision did not affect postoperative pain. As in UVATS all surgical maneuvers are performed through the same incision, a larger UVATS facilitates the exposure and resection of anatomical structures. Furthermore, it is easier to insert a surgical instrument into the chest cavity, resulting in a reduction of the repeated movements of the instruments through the uniportal incision and their force against the thoracic wall. In several studies, it resulted in a reduction of injury to the intercostal nerve which is the main source of pain after UVATS.^10^, ^11^, ^12^ It has also been reported that post‐operative pain was measured in the pain area by the BPI,^13^, ^14^, ^15^ although both surgical procedures (UVATS) were performed through one intercostal level in the present study. Based on those results, postoperative pain and its interference on daily functions after the same surgical technique did not depend on the incision length. Yet, it has also been reported that the easier exposure of anatomical structures also reduced manipulation of the lung that is an additional source of thoracic pain.^16^, ^17^

Second, the postoperative outcome was similar in both groups and no significant difference was found in terms of length of hospital stay and/or complication rates. However, there was a significant reduction in postoperative time in the LI group. Prolonged anesthesia may have possible adverse cardiovascular effects in patients undergoing thoracic surgery, in particular those patients with chronic obstructive pulmonary disease or preoperative cardiovascular disease. Reducing the operative time could positively affect the cardiac and pulmonary consequences after a prolonged anesthesia.

Our results should be considered with caution due to the small number of patients enrolled in the study. Additional limitations are the lack of evaluation of chronic pain and the aesthetic results of the incision.

In summary, a larger incision for UVATS significantly reduced the operative time due to better exposure of the anatomical structures without increasing postoperative pain or affecting the surgical outcome. Thus, to perform a larger incision could be a valuable strategy, particularly in nonexpert hands or when the patient's anatomy or tumor size make exposure of anatomic structures difficult through smaller incisions. Our preliminary results should be confirmed by a larger study in the future.

## Disclosure

The authors disclose no conflict of interest and no funding for this report.
